# Discrepancies in perception of fall risk between patients with subacute stroke and physical therapists in a rehabilitation hospital: a retrospective cohort study

**DOI:** 10.3389/fragi.2023.1204488

**Published:** 2023-06-05

**Authors:** Seigo Inoue, Yohei Otaka, Yukari Horimoto, Hidehiko Shirooka, Masafumi Sugasawa, Kunitsugu Kondo

**Affiliations:** ^1^ Department of Rehabilitation Medicine, Tokyo Bay Rehabilitation Hospital, Chiba, Japan; ^2^ Department of Rehabilitation Medicine I, School of Medicine, Fujita Health University, Toyoake, Aichi, Japan; ^3^ Education and Management in Health and Welfare Section, Health Sciences Program, Graduate School of International University of Health and Welfare, Tokyo, Japan

**Keywords:** accidental falls, cerebrovascular disorder, patient safety, rehabilitation, risk assessment, judgment

## Abstract

**Objective:** Falls are one of the most common complications of a stroke. This study aimed to clarify the discrepancy between the perceived fall risk of hospitalized patients with stroke and the clinical judgment of physical therapists and to examine the changes in discrepancy during hospitalization.

**Design:** Retrospective cohort study.

**Patients:** This study included 426 patients with stroke admitted to a Japanese convalescent rehabilitation hospital between January 2019 and December 2020.

**Methods:** The Falls Efficacy Scale-International was used to assess both patients’ and physical therapists’ perception of fall risk. The difference in Falls Efficacy Scale-International scores assessed by patients and physical therapists was defined as the discrepancy in fall risk, and its association with the incidence of falls during hospitalization was investigated.

**Results:** Patients had a lower perception of fall risk than physical therapists at admission (*p* < 0.001), and this trend continued at discharge (*p* < 0.001). The discrepancy in fall risk perception was reduced at discharge for non-fallers and single fallers (*p* < 0.001), whereas the difference remained in multiple fallers.

**Conclusion:** Unlike physical therapists, patients underestimated their fall risk, especially patients who experienced multiple falls. These results may be useful for planning measures to prevent falls during hospitalization.

## 1 Introduction

Falls are one of the most common complications of a stroke (Langhorne et al, 2000); approximately 5%–38% of patients fall at least once during their hospital stay (Nyberg and Gustafson, 1995; Teasell et al, 2002; Czernuszenko and Członkowska, 2009; Schmid et al, 2010; Persson et al, 2018). In particular, the fall rate among patients admitted to rehabilitation hospitals is high (Nyberg and Gustafson, 1995; Teasell et al, 2002; Czernuszenko and Członkowska, 2009). Falls can lead to adverse events such as fractures and head injuries (Schwendimann et al, 2008), prolonged hospitalization (Morello et al, 2015; Wong et al, 2016), and increased hospitalization costs (Morello et al, 2015). It is necessary to correctly identify the patients’ risk of fall and implement appropriate fall prevention measures.

Various risk factors for falls have been reported in patients with stroke admitted to rehabilitation hospitals, including the severity of motor impairment, impaired balance, cognitive function, urinary incontinence, number of medications, and previous fall history (Nyberg and Gustafson, 1997; Teasell et al, 2002; Nakagawa et al, 2008; Czernuszenko and Członkowska, 2009). Based on these findings, a number of screening assessment methods have been developed for the early detection of patients at high risk of falls (Nakagawa et al, 2008; Breisinger et al, 2014). Intuitive fall assessment by healthcare professionals, which is not limited to patients with stroke, has also been reported to have high fall prediction accuracy (Haines et al, 2007; Haines et al, 2009). A multicenter prospective study showed that physical therapists’ intuitive judgments of fall risk were effective in predicting falls in geriatric inpatients in a rehabilitation unit (Haines et al, 2009). A meta-analysis has also shown that nurses’ intuitive judgments of fall risk are equally good (Haines et al, 2007).

Fear of falling is associated with a risk of falling in patients with stroke and in older people (Jalayondeja et al, 2014; Freiberger et al, 2022). The Fall Efficacy Scale-International (FES-I) is a measure of patients’ own assessment of their fall risk (Yardley et al, 2005), and its score has been shown to be associated with fall occurrence in patients with stroke (Azad et al, 2014; Faria-Fortini et al, 2021). However, it has been pointed out that some patients may not be aware of their own risk of falls. In previous studies, hospitalized older patients with various diseases admitted to acute care hospitals (Lim et al, 2018) and outpatients with multiple sclerosis (Gunn et al, 2018) have reported discrepancies between their concern about fall risk and objective physiological fall risk. Similarly, in a study of patients with various diseases admitted to an acute care hospital, approximately half of the patients did not perceive themselves to be at high risk for falls, even when nurses assessed them as high risk (Twibell et al, 2015).

A difference in the perception of fall risk between patients and healthcare professionals may indicate that the patients themselves do not correctly perceive the risk of falls. In this situation, the risk of falls may increase due to misjudgments and actions beyond the acceptable range of one’s ability. However, to the best of our knowledge, no studies have examined the discrepancy in the perception of fall risk between patients with stroke admitted to rehabilitation hospitals and healthcare professionals. In addition, no studies have clarified the changes in the perception of fall risk over time or the relationship with falls.

This study aimed to determine the discrepancy in the perception of fall risk between therapists and patients with stroke admitted to a rehabilitation hospital, its change over time, and its association with the incidence of falls.

## 2 Materials and methods

### 2.1 Study design and participants

The study protocol was approved by the Institutional Review Board of Tokyo Bay Rehabilitation Hospital (approval number: 219-2). The study consisted of two parts. The main study was a retrospective study conducted to examine the association between differences in FES-I perception and falls. Participants included consecutively enrolled patients with stroke admitted to Tokyo Bay Rehabilitation Hospital from 1 January 2019, to 31 December 2020. The inclusion criterion was that the patients had a first-ever stroke. Exclusion criteria were difficulty obtaining data by interview due to aphasia or cognitive decline, and missing data. The requirement of informed consent was waived because of the retrospective study design; individuals who did not opt out were included.

In addition to the retrospective study, we conducted a reliability study to examine the intra- and inter-rater reliability of the FES-I assessment from the physical therapist’s perspective, which had been already used routinely at our hospital to determine a patient’s risk of fall. Participant consent procedures were performed in accordance with the Declaration of Helsinki, and written informed consent was obtained from all participants, both patients and evaluators (therapists).

The study results were reported according to the Strengthening Reporting of Observational Studies in Epidemiology reporting guidelines.

### 2.2 Fall prevention

The hospital has implemented falls prevention measures. The individual risk of falling was assessed regularly among all patients, and necessary countermeasures were implemented, such as using a wrist band for high-risk patients, supervision for/assistance in transfers/toileting/mobility, and appropriate environmental settings including mobility aids. Additionally, information on fall prevention during hospitalization was also provided in the form of pamphlets to educate patients and their families.

### 2.3 Data collection

Demographic information and patient characteristics, including age, sex, stroke type, paretic side, length of hospital stay, history of falls during hospitalization, FES-I score (Yardley et al, 2005), Functional Independence Measure (FIM) score (Keith et al, 1987), and Stroke Impairment Assessment Set-motor function (SIAS-m) score (Chino et al, 1994) were collected from patients’ medical records. FES-I, FIM, and SIAS-m scores were collected at admission and discharge, while other data were collected only at admission.

For the primary outcome, perception of fall risk, we employed the FES-I (Yardley et al, 2005). In the evaluation method, patients were interviewed regarding their anxiety about falling while performing 16 activities, which were scored on a 4-point Likert scale (1, not at all concerned; 4, very concerned; 16–64 total points), with higher scores indicating greater fear of falls. The validity of the FES-I has been confirmed in patients with stroke (Azad et al, 2014; Faria-Fortini et al, 2021). The same scale was used to assess the patient’s FES-I score from the perspective of the physical therapist. The evaluator was the patient’s physical therapist, who rated each item of the FES-I by replacing it with “how much concern do you think there would be about falling” if the patient performed that movement. In addition, FIM and SIAS-m were employed to assess the changes in physical function and activities of daily living (ADL) status during hospitalization.

ADL was measured using the FIM, which was assessed by trained nurses. The FIM consists of 13 motor subscales (FIM motor) and 5 cognitive subscales (FIM cognitive). Items on the scale are rated on a 7-point scale, with 1 representing complete dependence and 7 representing complete independence (Keith et al, 1987). The reliability and validity of this measure have been confirmed in patients with stroke (Ottenbacher et al, 1996).

The severity of motor paresis was assessed using the total SIAS-m (Chino et al, 1994). The upper limb motor score consists of two tests of the proximal and distal joints of the upper limb (0-12 points), and the lower limb motor score consists of three tests of the hip, knee, and ankle (0-15 points). The higher the score, the better the function. Its reliability and validity have been confirmed in patients with stroke (Chino et al, 1994; Tsuji et al, 2000).

### 2.4 Analyses

Patients were divided into three groups according to their history of falls during hospitalization: non-fall, single-fall, and multiple-fall groups. Descriptive statistics of the population characteristics were calculated. The Shapiro-Wilk test followed by the chi-square test and Kruskal-Wallis test were used to compare patient characteristics among the groups.

The within-group comparisons of FES-I scores were conducted using the Wilcoxon signed-rank test between patients and physical therapists and between admission and discharge. Discrepancies in the perception of fall risk were calculated as the difference between the FES-I assessed by the physical therapist and the value assessed by the patient. A positive value indicated that the patient perceived a lower risk of falls than the physical therapist. Within-group comparisons of the discrepancies between admission and discharge were performed using the Wilcoxon signed-rank test. Between-group comparisons of the discrepancies were performed using analysis of covariance (ANCOVA) with these variables as covariates because the number of days of hospitalization differed significantly between groups and because age and gender are potential confounders. When the ANCOVA indicated significant differences among groups, we performed *post hoc* comparisons using Bonferroni correction. To investigate how the FES-I scores assessed by patients or physical therapists related to motor paresis and ADL, correlations between FES-I scores and SIAS-m scores and between FES-I scores and FIM scores were examined using Spearman’s rank correlation coefficients on admission and at discharge, respectively.

Regarding the reliability study, intra-rater and inter-rater reliability of the therapist-assessed FES-I was examined in 40 stroke patients (patients’ characteristics are presented in Supplementary Table S1). The FES-I was re-assessed in each patient within 1 week of the initial assessment by the same therapist. Occupational therapists rated the FES-I scores in addition to physical therapists at the first assessment. The intraclass correlation coefficient (ICC) 1.1 and ICC 2.1 were used for intra-rater and inter-rater reliability, respectively. The ICC value was interpreted as follows: 0.0–0.5 poor; 0.5–0.75 moderate; 0.75–0.9 good; 0.9–1.0 excellent reliability (Koo and Li, 2016).

All data analyses were performed using STATA/BE 17 (StataCorp., College Station, Texas, United States). *p*-values less than 0.05 were considered statistically significant.

## 3 Results

**FIGURE 1:**
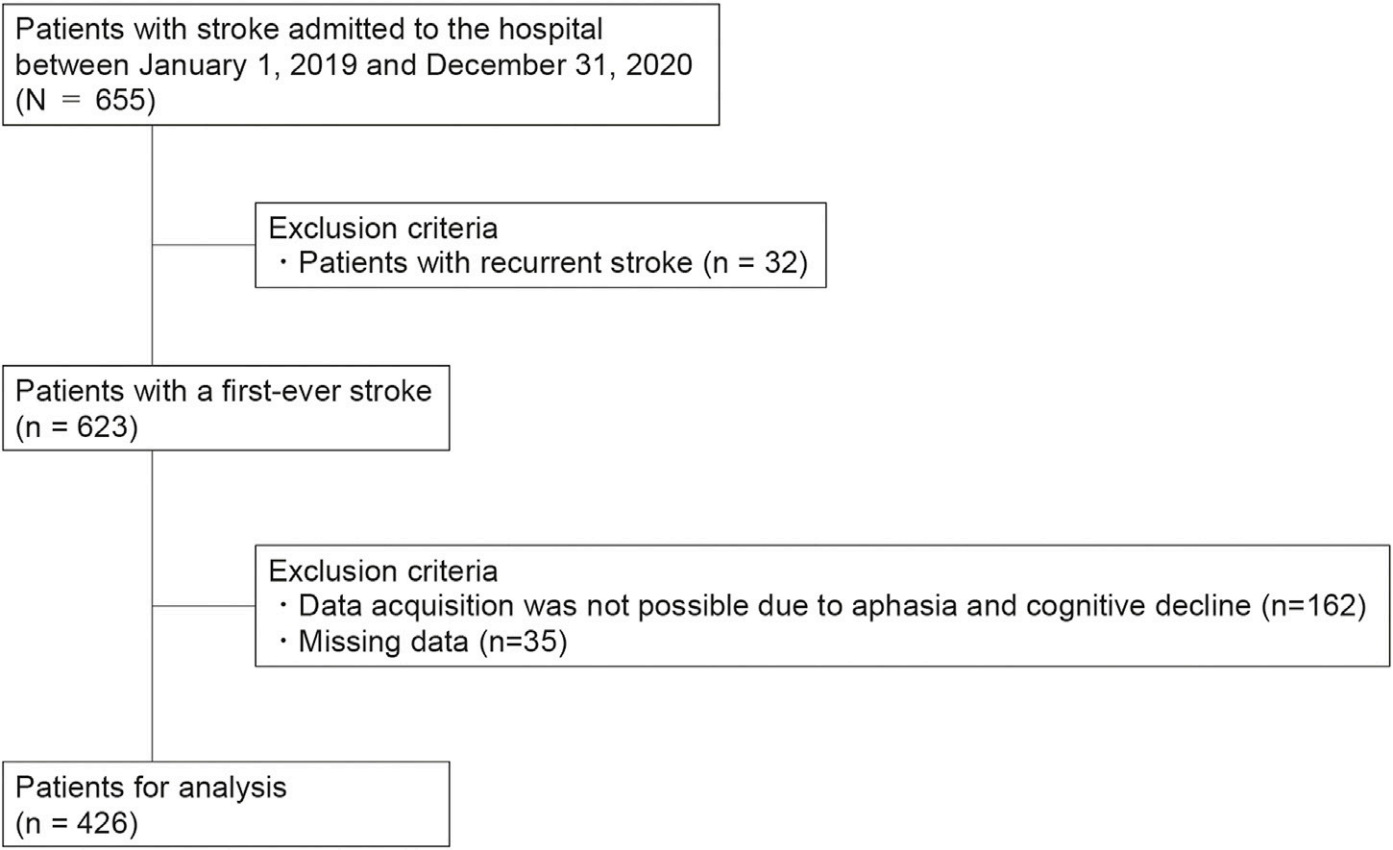
Flowchart of the patient selection process.

Among 655 consecutively enrolled patients with stroke, 426 met the inclusion and exclusion criteria and were analyzed (Figure 1).

Table 1 summarizes the characteristics of patients in each group at admission. The median (interquartile range) age of all participants (*n* = 426) was 71.0 (20.0) years, and the mean hospital stay was 79.0 (75.8) days. A total of 119 patients (27.9%) experienced one or more falls during hospitalization. Among them, 43 patients experienced multiple falls. The total number of falls was 200. The single- and multiple-fall groups had significantly longer hospital stays than the non-fall group.

**TABLE 1:** Participant characteristics.

Characteristics	Non-fall group	Single-fall group	Multiple-fall group	*p*-value
	(*n* = 307)	(*n* = 76)	(*n* = 43)	
Sex, male, n, (%)	202 (65.6)	39 (51.3)	29 (67.4)	0.054
Age, years, median (IQR)	71.0 (21.0)	75.0 (17.8)	71.0 (15.0)	0.097
Stroke type, hemorrhage/infarction, n	114/193	34/42	17/26	0.474
Affected side, right, n, (%)	169 (54.9)	37 (48.7)	19 (44.2)	0.300
Number of days of hospitalization, median (IQR)	64.0 (62.0)	109.0 (70.8)	133.0 (31.5)	<0.001*

IQR, interquartile range.

*Bonferroni’s *post hoc* results showed that the single- and multiple-fall groups had significantly longer hospital stays than the non-fall group.

Table 2 shows the clinical data at admission and discharge for each group. The severity of paralysis at both admission and discharge was significantly different between groups, with the multiple-fall group being the worst. The total FIM scores showed significant differences between the groups on admission, with the multiple-fall group showing the lowest values. At discharge, there were no significant differences between the fall groups.

**TABLE 2:** Clinical data at admission and discharge.

		Non-fall group	Single-fall group	Multiple-fall group	Group effect		Post-hoc test		
					F-value	*p*-value	Non-fall group vs. Single-fall group	Non-fall group vs. Multiple-fall group	Single-fall group vs. Multiple-fall group
SIAS U/E motor score, median (IQR)	Admission	10.0 (4.0)	9.0 (9.0)	3.0 (6.5)	41.05	<0.001	<0.001	<0.001	<0.001
	Discharge	11.0 (2.0)	10.0 (6.0)	5.0 (8.0)	39.09	<0.001	<0.001	<0.001	<0.001
SIAS L/E motor score, median (IQR)	Admission	13.0 (4.0)	11.5 (8.0)	5.0 (7.0)	49.42	<0.001	<0.001	<0.001	<0.001
	Discharge	15.0 (3.0)	12.0 (7.0)	9.0 (7.0)	46.79	<0.001	<0.001	<0.001	<0.001
SIAS-motor total score, median (IQR)	Admission	23.0 (7.0)	19.5 (16.0)	9.0 (12.5)	49.63	<0.001	<0.001	<0.001	<0.001
	Discharge	25.0 (5.0)	22.0 (13.0)	14.0 (12.0)	45.40	<0.001	<0.001	<0.001	<0.001
FIM motor score, median (IQR)	Admission	58.0 (24.0)	42.5 (20.3)	32.0 (18.5)	53.04	<0.001	<0.001	<0.001	0.018
	Discharge	87.0 (9.0)	78.5 (26.0)	72.0 (20.5)	25.94	<0.001	<0.001	<0.001	0.310
FIM cognitive score, median (IQR)	Admission	28.0 (9.0)	23.0 (13.0)	22.0 (13.5)	14.46	<0.001	<0.001	<0.001	0.999
	Discharge	32.0 (8.0)	30.0 (9.0)	29.0 (6.5)	5.53	0.004	0.011	0.129	0.999
FIM total score, median (IQR)	Admission	85.0 (31.0)	67.5 (28.8)	55.0 (27.0)	48.13	<0.001	<0.001	<0.001	0.040
	Discharge	117.0 (15.0)	107.5 (32.8)	100.0 (25.5)	22.24	<0.001	<0.001	<0.001	0.657

IQR, interquartile range; SIAS, stroke impairment assessment set; U/E, upper extremity; L/E, lower extremity; FIM, functional independence measure.

**FIGURE 2:**
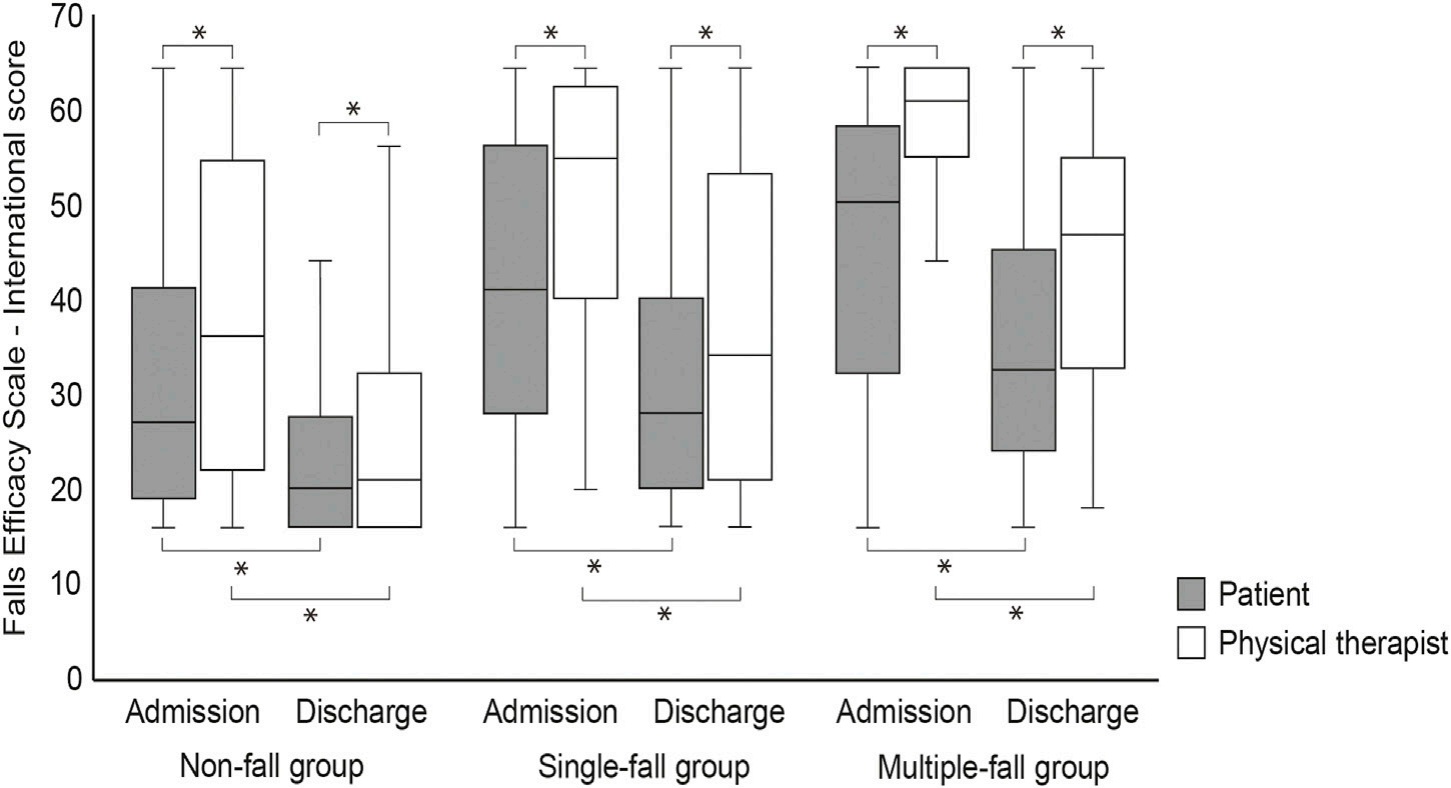
Falls Efficacy Scale-International at admission and discharge as assessed by patients and physical therapists *p < 0.05.

Figure 2 shows the results of the FES-I for each group as assessed by the patients and physical therapists on admission and discharge. The median (interquartile range) FES-I score assessed by the patient and by physical therapists on admission was 27.0 (22.0) and 36.0 (32.5), respectively, for the non-fall group; 41.0 (27.3) and 55.0 (22.3), respectively, for the single-fall group; and 50.0 (25.5) and 61.0 (8.5), respectively, for the multiple-fall group. The FES-I assessed by patients and physical therapists at discharge was 20.0 (12.0) and 22.0 (16.0), respectively, in the non-fall group, 28.0 (19.3) and 34.0 (32.3), respectively, in the single-fall group, and 33.0 (19.5) and 47.0 (21.0), respectively, in the multiple-fall group. The FES-I scores assessed by patients as well as physical therapists improved significantly at discharge compared to that at admission. The FES-I scores assessed by patients were significantly lower than those assessed by physical therapists, indicating that patients perceived a lower risk of falls than physical therapists.

**FIGURE 3:**
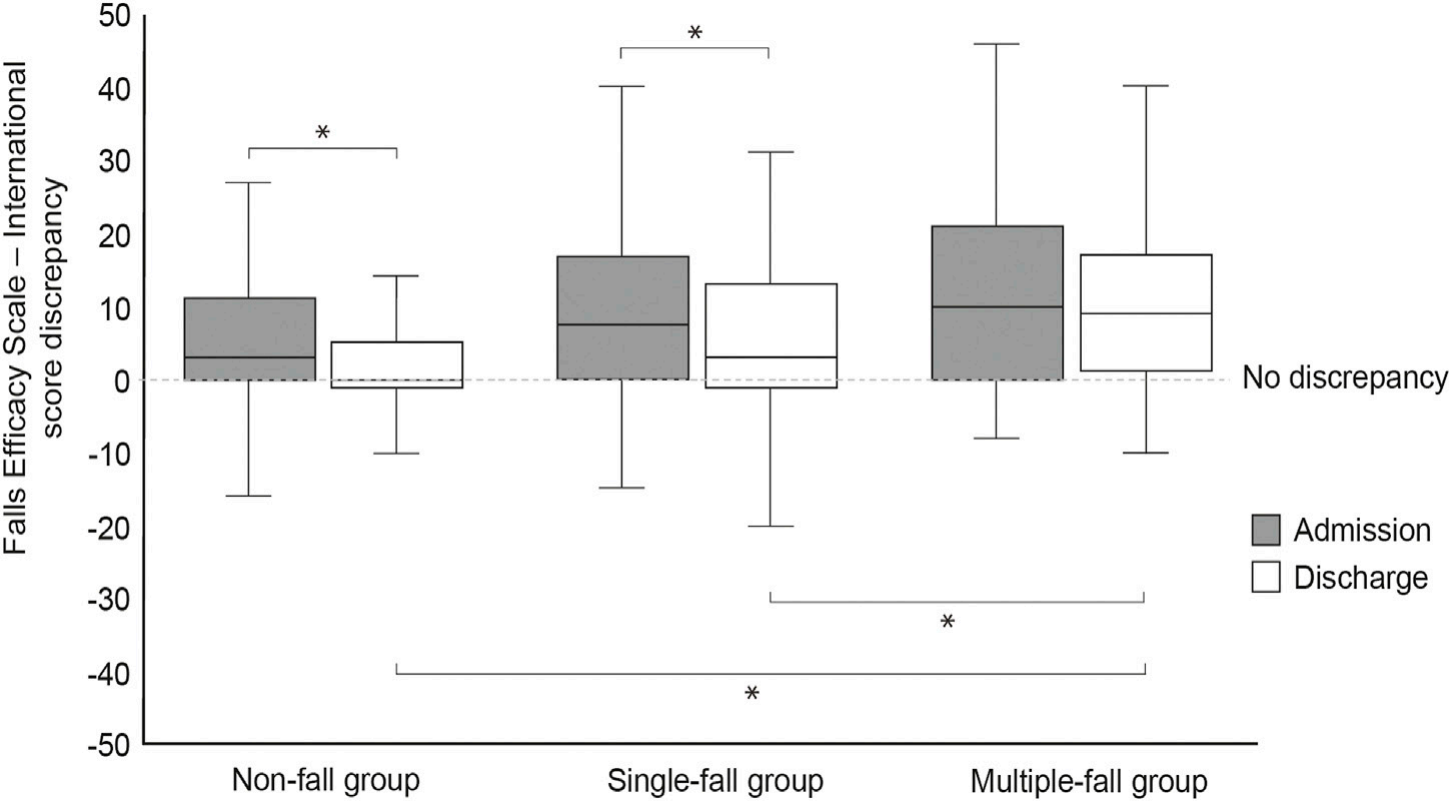
Discrepancies in the perception of fall risk between admission and discharge in each group *p < 0.05.

Figure 3 shows the discrepancy in perception of fall risk on admission and discharge for each group. Discrepancies in fall perception on admission and at discharge were median 3.0 (interquartile range 11.0) and 0.0 (6.0), respectively, in the non-fall group; 7.5 (16.3) and 3.0 (14.0), respectively, in the single-fall group; and 10.0 (21.0) and 9.0 (16.0), respectively, in the multiple-fall group. The discrepancy between the perception of fall risk between patients and physical therapists was significantly reduced from admission to discharge in the non-fall and single-fall groups, while the discrepancy was not reduced in the multiple-fall group. In the between-group comparisons of discrepancies in perception of fall risk, ANCOVA with number of days of hospitalization, age, and sex as covariates showed no significant group effects at admission (*p* = 0.092). There was a between-group effect at discharge (*p* = 0.011), and *post hoc* test results showed that the multiple-fall group was significantly larger than the non-fall and the single-fall group.

Table 3 shows the correlations between the total scores of the FES-I, SIAS-motor, and FIM as assessed by the patient and the physical therapist at admission and discharge. Each showed a significant relationship, with correlation coefficients higher for the results assessed by the physical therapist.

**TABLE 3:** Relationship between the FES-I and each variable as assessed by the patient and the physical therapist.

			SIAS-motor scores			FIM total scores		
			rho	(95% CI)	*p*-value	rho	(95% CI)	*p*-value
FES-I	Patients	Admission	−0.491	(−0.560 to −0.415)	<0.001	−0.498	(−0.566 to −0.423)	<0.001
		Discharge	−0.400	(−0.477 to −0.317)	<0.001	−0.583	(−0.642 to −0.517)	<0.001
	Physical therapists	Admission	−0.606	(−0.663 to −0.542)	<0.001	−0.767	(−0.803 to −0.725)	<0.001
		Discharge	−0.437	(−0.511 to −0.357)	<0.001	−0.755	(−0.793 to −0.711)	<0.001

SIAS, stroke impairment assessment set; FIM, functional independence measure; FES-I, falls efficacy scale-international; CI, confidence interval.

The intra-rater reliability (ICC 1.1) of the FES-I score rated by physical therapists was excellent at 0.997 (95% CI, 0.994 to 0.998; *p* < 0.001). Inter-rater reliability (ICC 2.1) between physical and occupational therapists was also good at 0.812 (95% CI, 0.673 to 0.896, *p* < 0.001).

## 4 Discussion

The study revealed the difference in perceptions of fall risk between physical therapists and patients with stroke admitted to a rehabilitation hospital and its relationship to falls. At both admission and discharge, patients perceived their risk of falls to be lower than that perceived by physical therapists. Discrepancies in the perception of fall risk were significantly reduced at discharge compared to those at admission in the non-fall and single-fall groups, but not in the multiple-fall group.

Previous studies examining differences in fall risk between patients and healthcare workers have used different scales (Twibell et al, 2015; Gunn et al, 2018; Lim et al, 2018), which makes it difficult to show the extent of the differences in perception between patients and healthcare providers. In this study, we assessed the fall risk from the perspective of patients and healthcare workers using the same FES-I scale. Since the FES-I is a scale that originally reflects the psychological aspects of the patients themselves, we checked the reliability of other people’s evaluations and found that the intra- and inter-examiner reliability was excellent. Interestingly, the FES-I, as assessed by the physical therapist, correlated more strongly with the SIAS-motor and FIM total scores than with the patient’s assessment, indicating the FES-I assessed by a physical therapist might better reflect actual movement ability.

A study examining changes in fall-related self-efficacy over time in hospitalized patients with stroke reported improvements in fall-related self-efficacy as their abilities improved (Hellström et al, 2003). On the other hand, it is not clear how fall-related self-efficacy changes in patients with stroke who experience a fall during hospitalization. This study clearly indicates that, regardless of their history of falls, patients with stroke have improved fall-related self-efficacy at discharge compared to that on admission, which may be because the fallers also showed improvement in physical function and ADL during the course of hospitalization. In contrast to our findings, a study that examined changes in fall-related self-efficacy over time in community-dwelling elderly people reported a gradual decline regardless of the history of falls, with a greater decline seen in those who had fallen multiple times (Delbaere et al, 2010). These findings indicate that the perception of falls changes positively with the recovery of physical function in patients with stroke regardless of falls, and the impact of falls may not be consistent with findings in older adults.

The present study suggested that patients with falls overestimated their fall self-efficacy. In addition, the discrepancy in fall risk perception in the single-fall group decreased at the time of discharge from the hospital compared to that at the time of admission, but no change was observed in the multiple-fall group. Reportedly, some falls in hospitalized stroke patients are caused by movements beyond the permitted range based on the patient’s own judgment (Hanger et al, 2014). In a previous study (Walsh et al, 2019) that interviewed people who had repeated falls in the first year after stroke, patients made statements such as prioritizing activities despite being at a risk for falls. Similarly, in another study (Aihara et al, 2021), patients with stroke who experienced a fall during hospitalization were interviewed within a week after the fall; overconfidence in ability, lack of awareness, and lack of anticipation of the fall were cited as causes of the fall, suggesting that stroke patients do not correctly judge their fall risk. In the present study, there was no change in the discrepancy in the perception of fall risk between admission and discharge in the multiple-fall group, suggesting that their inappropriate perception of their fall risk contributed to the recurrence of falls.

The study was conducted in the setting where fall prevention measures were implemented. Therefore, the findings obtained in the present study were influenced by the implementation of counter measures for fall prevention. Especially, education on fall prevention might affect to decrease the discrepancy in patients’ perceptions of in fall risk. It is very interesting to note that, despite this situation, the discrepancy in their perceptions of fall risk remained in the multiple-fall group. In other words, the results of this study pointed that the fall prevention provided in the setting of the present study was insufficient or ineffective for patients who have a large discrepancy in their perceptions of fall risk. Future studies are required to determine whether more rigorous strategies, such as repeated one-on-one therapist education (Haines et al, 2011; Hill et al, 2015), would modify the discrepancy in fall risk perception in patients at high fall risk.

A limitation of this study is that only the history of falls during hospitalization was considered in the present study, as it was difficult to accurately obtain the history of falls prior to hospitalization. Also, its retrospective study design in a single hospital. The generalizability of patients in other institutions cannot be warranted. However, the fall rate of 0.47 per patient (200 falls in 426 patients) in the present study was comparable to the fall rates ranging from 0.22 to 0.95, as reported in previous studies (Nyberg and Gustafson, 1995; Teasell et al, 2002; Czernuszenko and Członkowska, 2009). Furthermore, the characteristics of the fallers in the present study, that is, more severe paralysis and lower ADL, were similar to those observed in previous studies (Nyberg and Gustafson, 1995; Teasell et al, 2002; Czernuszenko and Członkowska, 2009). Considering that the stroke patients included in the present study had similar characteristics to those in previous studies, the findings of the present study may have certain generalizability. A large multicenter study will confirm the findings of this study.

## 5 Conclusion

This study revealed that the FES-I assessed by stroke patients differed from that assessed by physical therapists and that the extent of the difference was related to the incidence of falls. The study also revealed that the FES-I did not worsen with the experience of falling but improved as the ability improved. Furthermore, our results suggested that those who fall multiple times may have overestimated their abilities more than the objective viewpoint of a physical therapist.

## Data Availability

The data analyzed in this study is subject to the following licenses/restrictions: The datasets used and/or analyzed during the current study are available from the corresponding author on reasonable request. Requests to access these datasets should be directed to YO, otaka119@mac.com.
