# Supplementary material for: Discrepancies in perception of fall risk between patients with subacute stroke and physical therapists in a rehabilitation hospital: a retrospective cohort study
Source: Front Aging. 2023 Jun 5;4:1204488. doi: 10.3389/fragi.2023.1204488 (PMC10277567; doi:10.3389/fragi.2023.1204488)
Supplement: Supplementary file 1 [file Table1.DOCX]

Supplementary Material

Discrepancies in perception of fall risk between patients with subacute stroke and physical therapists in a rehabilitation hospital: A retrospective cohort study

Seigo Inoue^1^, Yohei Otaka^1,2*^, Yukari Horimoto^3^, Hidehiko Shirooka^3^, Masafumi Sugasawa^1^, and Kunitsugu Kondo^1^

*** Correspondence:** Yohei Otaka, E-mail: otaka119@mac.com

**Supplementary Tables.** Participant characteristics for the reliability study

|  | n = 40 |
| --- | --- |
| Sex, male/female, n | 24/16 |
| Age, years, mean (SD) | 66.3 (13.2) |
| Days after onset (day) ^†^ | 96.6 (49.7) |
| Length of stay (days) ^†^ | 60.2 (42.4) |
| Affected side, right/left, n | 20/20 |
| SIAS lower limb motor score, median (IQR) | 10.5 (3.3) |
| FIM, motor score, median (IQR) | 73.0 (36.0) |
| FIM, cognition score, median (IQR) | 30.0 (9.0) |
| FIM, total score, median (IQR) | 102.0 (39.0) |

To clarify the reliability of the FES-I by others, 40 patients with stroke were included, separate from the present study. Abbreviations: SD, standard deviation; IQR, interquartile range; SIAS, Stroke Impairment Assessment Set; FIM, Functional Independence Measure; FES-I, Fall Efficacy Scale-International.
